# Association between opioid abuse and COVID-19 susceptibility: a propensity score matched study

**DOI:** 10.1186/s12879-023-08842-4

**Published:** 2023-12-05

**Authors:** Mojtaba Hedayatyaghoobi, Mehdi Azizmohammad Looha, Arman Shafiee, Kyana Jafarabady, Omid Safari, Amirhesam Alirezaei, Mahmood Bakhtiyari

**Affiliations:** 1 Department of Infectious Diseases, Alborz University of Medical Sciences, Karaj, Iran; 2 Basic and Molecular Epidemiology of Gastrointestinal Disorders Research Center, Research Institute for Gastroenterology and Liver Diseases, Shahid Beheshti University of Medical Sciences, Tehran, Iran; 3 Department of Psychiatry and Mental Health, Alborz University of Medical Sciences, Karaj, Iran; 4 Student Research Committee, School of Medicine, Alborz University of Medical Sciences, Karaj, Iran; 5 Non-Communicable Diseases Research Center, Alborz University of Medical Sciences, Karaj, Iran; 6 Department of Nephrology, Shahid Beheshti University of Medical Sciences, Tehran, Iran; 7 Non-Communicable Diseases Research Center, Alborz University of Medical Sciences, Karaj, Iran

**Keywords:** COVID-19, Opioid, Abuse, Propensity score, Mortality

## Abstract

**Background:**

Opioid use disorder (OUD) has been associated with adverse health outcomes, and its potential impact on COVID-19 outcomes is of significant concern. This study aimed to assess the susceptibility and clinical outcomes of hospitalized COVID-19 patients with OUD using a propensity score-matched design.

**Methods:**

A historical cohort study was conducted in Alborz province, Iran, during the early months of the COVID-19 pandemic. Patients aged 18 years and above with confirmed COVID-19 were included in the study. OUD was defined as a compulsive urge to use opioids or opioid-derivative drugs. Non-opioid abusers with COVID-19 were selected as the control group. Data on demographics, clinical characteristics, laboratory factors, comorbidities, and vital signs were collected. Propensity score matching (PSM) was used to balance the groups and assess the impact of OUD on ICU admission, mortality, the need for intubation, and the severity of pulmonary involvement on CT scans.

**Results:**

A total of 442 patients were included in the study, with 351 discharged and 34 deceased. The PSM analysis showed that OUD was not significantly associated with ICU admission (OR: 1.87, 95% CI: 0.22–2.91, *p* = 0.631). However, opium users had an increased risk of mortality (OR: 2.38, 95% CI: 1.30–4.35, *p* = 0.005) and a higher likelihood of requiring intubation (OR: 3.57, 95% CI: 1.38–9.39, *p* = 0.009) compared to non-opioid abusers. The severity of pulmonary involvement on CT scans did not show a significant association with OUD.

**Conclusion:**

OUD among hospitalized COVID-19 patients was associated with an increased risk of mortality and the need for intubation. These findings highlight the importance of addressing OUD as a potential risk factor in the management and treatment of COVID-19 patients. Further research is warranted to explore the underlying mechanisms and develop appropriate interventions to mitigate the impact of OUD on COVID-19 outcomes.

**Supplementary Information:**

The online version contains supplementary material available at 10.1186/s12879-023-08842-4.

## Introduction

The COVID-19 pandemic, caused by the novel coronavirus SARS-CoV-2, has emerged as a global health crisis affecting millions worldwide [1]. The virus has presented a wide range of clinical symptoms, including mild respiratory symptoms, severe pneumonia, and even fatal outcomes [2]. While several risk factors associated with COVID-19 severity and mortality have been identified, the potential impact of prior opium addiction on the disease's course and outcomes remains an area of considerable interest and concern [3].

Opium addiction, a significant public health problem in many regions, poses unique challenges in the context of COVID-19 [4]. Opium, which is derived from the poppy plant, contains alkaloids such as morphine and codeine that exert powerful effects on the central nervous system [5]. The addictive properties of opium and its derivatives have been well-documented, with profound implications for individuals' physical and mental well-being [6]. Iran, the region of current study, has the highest rate of opium users with 2.7% of the population [7, 8].

Previous research indicates that opium addiction can lead to various systemic health consequences, including respiratory complications, immunosuppression, and increased susceptibility to infections [9–11]. Given the respiratory tropism of SARS-CoV-2 and the potential for opium-related respiratory impairments, it is crucial to investigate the impact of prior opium addiction on COVID-19 outcomes.

Understanding the potential association between opium addiction and COVID-19 outcomes has significant implications for public health strategies, clinical management, and harm reduction interventions [12]. Specifically, elucidating the interplay between opium addiction and COVID-19 can guide healthcare providers in risk assessment, treatment planning, and resource allocation, ultimately optimizing patient care and reducing the burden on healthcare systems [13].

Limited studies have specifically examined the relationship between opium addiction and COVID-19 outcomes. Existing evidence suggests that opium addiction may be associated with worsened COVID-19 outcomes, including increased disease severity, higher intensive care unit (ICU) admission rates, and elevated mortality risk [14]. However, the available studies are limited in sample size, geographical representation, and comprehensive analysis of potential confounding factors. Therefore, this research article aims to comprehensively analyze the impact of prior opium addiction on COVID-19 outcomes, considering critical factors such as age, gender, comorbidities, socioeconomic status, and healthcare access. By conducting a systematic review and meta-analysis of available literature, we will consolidate existing evidence and provide a comprehensive evaluation of the relationship between opium addiction and COVID-19 outcomes. This study’s findings can inform clinical decision-making, public health policies, and targeted interventions for individuals with a history of opium addiction during the COVID-19 pandemic. Additionally, this research will shed light on the underlying mechanisms through which opium addiction may influence COVID-19 outcomes, potentially paving the way for future studies exploring therapeutic interventions and harm reduction strategies. Ultimately, this study will contribute to enhancing our understanding of the complex interplay between opium addiction and COVID-19 outcomes, facilitating informed decision-making by healthcare providers and policymakers alike.

## Methods

### Study design

This study employed a historical cohort design to assess the risk of mortality, ICU admission, and need for intubation among hospitalized COVID-19 patients.

### Study setting

The investigation was carried out in the Alborz province, Iran, during the early months of the COVID-19 pandemic. The Alborz University of Medical Sciences Local Ethics Committee approved the study protocol under the IR code. COVID-19 confirmation was made through a positive polymerase chain reaction (PCR) result. Opium use disorder was defined as a compulsive urge to use opioids or opioid-derivative drugs. In this study, the recent definition was considered, which involved inhaling the smoke from burning dark crude material obtained from Papaver somniferum.

### Participants

Participants aged 18 years and above with confirmed COVID-19 were included in the study, as opium use disorder is rare among younger age groups. To form the control group, non-opioid abusers who contracted COVID-19 were included. These individuals were selected based on the absence of opioid use disorder (OUD), and they were matched with the opium-abusing group by age, gender, and other relevant demographic factors. In the current study “Opium” use is considered as a substance use disorder (SUD) which is defined as a mental disorder that has influence on patient’s brain and behavior, results in inability to control use of substances [15].

Patients who were transferred to other hospitals during the course of their treatment and individuals with missing data for any of the studied variables were excluded from the study. The exclusion of patients transferred to other hospitals was necessary because we could not follow their outcomes, potentially introducing variability. Excluding patients with missing data was essential to maintain dataset completeness and minimize potential bias resulting from incomplete information. It is important to note that the excluded patients were not substantially different from those who remained in the study in terms of the variables considered, thereby minimizing the risk of sampling bias.

### Variable

The study focused on three primary outcomes: mortality, ICU admission, and mechanical ventilation. The researchers contacted the families of deceased patients to verify the recorded outcomes and complete any missing information on the data collection forms. Additionally, if individuals were discharged from the hospital and gave consent, telephone follow-ups were conducted one or two weeks after discharge to gather further information. Secondary outcomes included the severity of CT scan involvement and O2 saturation.

Trained nurses conducted interviews to gather data on demographic characteristics, medical history, medication use, and smoking habits [16]. Venous blood samples were collected from participants after an overnight fast of 12–14 h, between 07:00 and 09:00 a.m. Centrifugation was performed within 30–45 min of sample collection. The serum total cholesterol (TC) level was measured using the enzymatic colorimetric method with cholesterol esterase and cholesterol oxidase. Other metabolic measurements such as high-density lipoprotein cholesterol (HDL-C) and triglyceride (TG) levels were determined using previously reported methods [17].

Participants were categorized as current smokers (those who were currently smoking) or non-smokers (those who had never smoked or had quit smoking). Regular opium use was identified if a participant had a history of chronic OUD (through respiratory or gastrointestinal routes) for at least one year and experienced withdrawal symptoms upon cessation of opium use. Weight measurements were taken with minimal clothing and without shoes, using digital scales (Seca 707; range 0.1–150 kg), and recorded to the nearest 100 g. Height was measured while participants were standing without shoes, with their shoulders in a normal alignment. Body mass index (BMI) was calculated by dividing the weight in kilograms by the square of the height in meters.

Type 2 diabetes (T2D) was defined as a fasting plasma glucose (FPG) level of ≥ 7.0 mmol/L and/or a 2-h post-challenge plasma glucose (PCPG) level of ≥ 11.1 mmol/L, or current use of anti-diabetic medications [18]. Fever was characterized as an axillary temperature of at least 37.3°C. Secondary infection was diagnosed when patients exhibited clinical symptoms or signs of pneumonia or bacteremia, and a new pathogen was identified through positive culture from lower respiratory tract specimens (qualified sputum, endotracheal aspirate, or bronchoalveolar lavage fluid) or blood samples after admission. Acute kidney injury was diagnosed according to the Kidney Disease: Improving Global Outcomes (KDIGO) clinical practice guidelines [19], while acute respiratory distress syndrome (ARDS) was diagnosed based on the Berlin Definition [20].

In this study, coronary heart disease (CHD) events were considered as outcomes and included cases of definite myocardial infarction (MI) diagnosed by electrocardiogram (ECG) and biomarkers, probable MI (positive ECG findings plus cardiac symptoms or signs, but biomarkers showing negative or equivocal results), unstable angina pectoris (new cardiac symptoms or changing symptom patterns with positive ECG findings and normal biomarkers), angiography-proven CHD, and CHD-related deaths (any death due to CHD based on the aforementioned criteria that occurred in the hospital or sudden cardiac death from cardiac disease within 1 h of symptom onset based on verbal autopsy documents outside the hospital).

### Data sources/measurement

To gather patients' demographic and clinical data, blood biochemical factors, and information related to COVID-19 signs, symptoms, treatment, and outcomes, a data collection form was developed and utilized. To ensure consistent distribution and completion of the forms, a series of three two-hour training sessions were conducted for 32 nurses from various hospitals in Alborz province, Iran, encompassing a total of 17 hospitals. Subsequently, the data collection teams within each hospital submitted the completed forms to the leading researchers of this study through an integrated electronic inter-sectoral system.

Data collection occurred during the admission phase, treatment period, and when the outcome (death or recovery) was observed. To maintain the quality and accuracy of the recorded information, continuous quality control of the completed questionnaires was implemented. As a first step, a subset of randomly selected questionnaires was assessed for item accuracy through telephone interviews with the responsible nurses. Throughout the data collection process, five individuals independently entered and coded the questionnaire responses using Excel software. Discrepancies were resolved by reviewing the questionnaire, contacting the study subjects, or excluding them from the study if necessary.

### Bias

To mitigate potential sources of bias in this study, several measures were implemented. First, the use of propensity score matching (PSM) was employed to balance the treatment and control groups, minimizing the impact of confounding variables and selection bias. This technique allowed for a more rigorous comparison of outcomes. Additionally, data collection quality was controlled continuously through a series of training sessions for 32 nurses from various hospitals. These training sessions aimed to ensure uniform data collection across multiple healthcare institutions and minimize variability in data collection. Furthermore, to address potential information bias, a subset of randomly selected questionnaires was reviewed for item accuracy through telephone interviews with the responsible nurses. Any discrepancies were resolved through rigorous data validation procedures, enhancing the accuracy and reliability of the recorded information.

### Study size

The study included a total of 442 participants. Given the retrospective cohort design, all eligible individuals meeting the inclusion criteria were incorporated into the study, and no specific sampling was conducted.

### Statistical analysis

Continuous baseline demographic and clinical data are presented as mean ± standard deviation and grouped data as frequencies and percentages. The comparison of means between the discharged and deceased groups, for quantitative variables with approximately normal distribution, was performed using independent t-tests. The distribution of non-normal quantitative data between the two groups was assessed using the exact Mann–Whitney U test. Chi-square test or Fisher’s exact test were used to determine the independence of the two categorical variables. To assess the treatment effect on the outcome variables, the PSM method was employed. Initially, potential confounding variables were identified. Logistic regression was then employed to determine the variables associated with the outcome or treatment, which were subsequently included in the model. For the ICU outcome, covariates included Age, Sex, Smoking, Alcohol, fever, Cancer, Ghalyan history, ARDS, BP, PLT, NUT, BUN, LDH, TG, Ventilator need, Time ventilation, Posterior Lower Lobe predication, Inpatients duration, ceftazidime, clindamycin, and vancomycin. For the mortality outcome, covariates included Smoking, Temperature, Age, Sex, Alcohol, ceftazidime, Ghalyan history, Ventilator need, INR, Time ventilation, PLT, BP, Cancer, vancomycin, Impatient, LDL, CRP, AST, FBS, Air bronchogram, BUN, LYMP, NUT, Bad breath, and linezolid. For the need to ventilation outcome, covariates included Age, Sex, Smoking, Alcohol, first sign time, Nasua, Cancer, Ghalyan history, ICU, ARDS, BP, GCS, PLT, LYMP, BUN, LDH, TG, AST, Ventilator need, Time ventilation, Posterior Lower Lobe predication, Inpatients duration, ceftazidime, amikacin, clindamycin, vancomycin, linezolid, and oseltamivir. PSM was conducted using the nearest neighbor algorithm, matching each individual in the treatment group with three individuals from the control group, resulting in a 1:3 matching ratio [21]. Various methods, including the absolute standardized mean difference (SMD) [22], variance ratio [23], and Kolmogorov–Smirnov (KS) statistic [24], were used to assess balance and achieve an optimal matched set that would provide a reliable estimation of the treatment effect after PSM. A good balance between the two groups was considered when the absolute SMD approached zero, the variance ratio approached one, and the KS statistic approached zero.

To estimate the hazard ratio (HR) and its standard error, a weighted proportional hazards Cox model with robust standard errors and clustering by subgroup was utilized. The weighted data were subsequently used to construct the Kaplan–Meier plot. Furthermore, for the analysis of treatment effect, both the full adjustment method, which involved adjusting for all variables in a logistic regression model alongside the treatment effect, and the optimal model selection method, utilizing the stepwise backward algorithm, were employed.

All statistical analyses were performed using the R software. Statistical significance was determined based on a significance level of *p* < 0.05.

## Results

### Patients’ characteristics and demographic variables

**Fig. 1:**
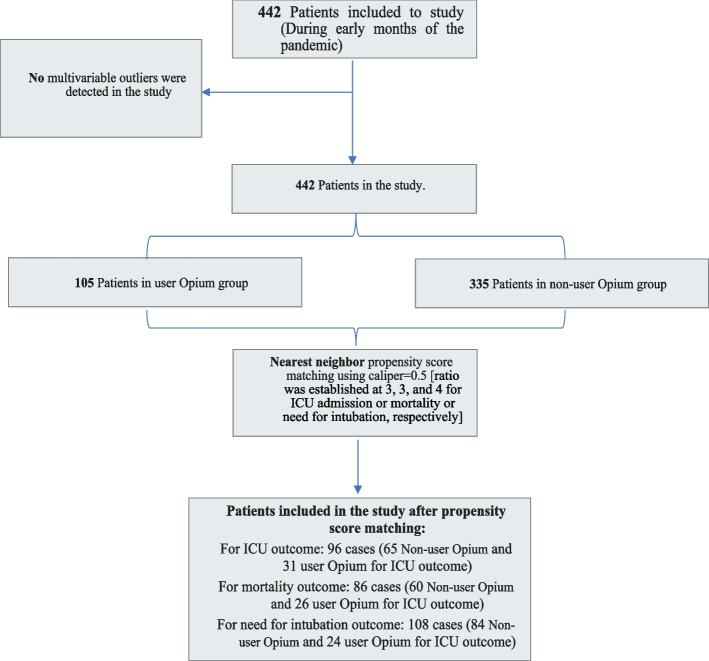
Flow chart of patient in the analysis

In this study, data from a total of 442 patients were included in the analysis. Among these patients, 351 were discharged from the hospital, and 34 were reported as deceased based on the available data. It is important to note that there were missing values for the vital status of a subset of patients, which may contribute to the discrepancy in the numbers provided. A graphical representation of patient characteristics can be found in Fig. 1. Out of the total 442 patients included in the study, 105 individuals (23.8%) were identified as Opium users, while 337 patients (76.2%) were classified as non-users. The variables analyzed included age, body mass index (BMI), waist circumference, sex, smoking status, and alcohol consumption. The mean and standard deviation of age was 75.52 ± 12.97 years. There was no significant difference in age between the discharged group (76.21 ± 12.41 years) and the deceased group (76.41 ± 13.03 years) (*p* = 0.930). In addition, the results indicated no significant differences in BMI (*p* = 0.506) between the two groups. However, waist circumference showed a significant difference (*p* < 0.001) with higher values observed in the deceased group. No significant associations were found between sex (*p* = 0.714), smoking status (*p* = 1.000), and alcohol consumption (*p* = 0.498) with the outcome variable (Table 1).

**Table 1:** The patients’ characteristics and demographic variables

Variable	Levels	Total (*n* = 442)	Outcome		*P*-value
			**Discharge (*n* = 351)**	**Deceased (*n* = 34)**	
Age	–––	75.52 ± 12.97	76.21 ± 12.41	76.41 ± 13.03	0.930^a^
BMI	–––	26.10 ± 4.52	26.14 ± 4.52	26.72 ± 5.03	0.506^a^
Waist	–––	82.00 ± 21.76	82.56 ± 21.07	40.00 ± 0.00	< 0.001^a^
Sex	Male	266 (60.18)	210 (59.83)	22 (64.71)	0.714^b^
	Female	174 (39.37)	139 (39.60)	12 (35.29)	
	NA	2 (0.45)	2 (0.57)	0 (0.00)	
Smoking	Non-smoker	341 (77.15)	278 (79.20)	27 (79.41)	1.000^b^
	Smoker	99 (22.40)	71 (20.23)	7 (20.59)	
	NA	2 (0.45)	2 (0.57)	0 (0.00)	
Alcohol consumption	Non-alcoholic	410 (92.76)	325 (92.59)	33 (97.06)	0.498^b^
	Alcohol use disorder	30 (6.79)	24 (6.84)	1 (2.94)	
	NA	2 (0.45)	2 (0.57)	0 (0.00)	

Quantitative variables are presented as mean ± standard deviation. Qualitative variables are reported as frequency (percentage). a) An independent t-test was utilized to compare the means of quantitative variables with an approximately normal distribution between the discharged and deceased groups. b) Fisher's exact test was applied to assess the association between qualitative variables and the outcome variable

### Sign and symptoms, laboratory factors, comorbidities and vital signs

#### Signs and symptoms

The mean temperature was significantly higher in the deceased group (38.20 ± 0.84) compared to the discharged group (36.72 ± 4.59), with a p-value of 0.009. No significant differences were observed between the groups in terms of the time of onset of the first symptom, number of individuals exposed to contact, and time interval from the first symptom to admission. Among the individual symptoms, halitosis showed a significant association with the outcome (*p* = 0.043), with a higher proportion of patients with halitosis in the deceased group (67.65%) compared to the discharged group (51.28%). No significant associations were found for other symptoms, including fever, cough, septum, sore throat, rhinorrhea, headache, muscle pain, loss of appetite, diarrhea, nausea, vomiting, and dizziness.

#### Laboratory findings

Several laboratory factors showed significant differences between the discharged and deceased groups. The deceased group had significantly higher levels of TPR (*p* < 0.001), PR (*p* = 0.001), RR (*p* < 0.001), WBC (*p* < 0.001), VITD (*p* = 0.021), FBS (*p* = 0.001), BUN (*p* < 0.001), CR (*p* < 0.001), CA (*p* = 0.002), LDH (*p* < 0.001), LDL (*p* = 0.008), TG (*p* = 0.003), AST (*p* < 0.001), ALT (*p* < 0.001) and lower levels of BP (*p* < 0.001), SPO2 (*p* < 0.001), PO2 (*p* = 0.001), GCS (*p* = 0.004), HB (*p* < 0.001), PLT (*p* < 0.001), ESR (*p* < 0.001), CRP (*p* < 0.001), PT (*p* = 0.001), PTT (*p* = 0.001), INR (*p* < 0.001), NA (*p* < 0.001), K (*p* < 0.001), and HDL (*p* = 0.005). No significant differences were found for DDIMER.

#### Comorbidities

No significant associations were found between outcome and comorbidities, including pregnancy, CVD, HBB, HBc, HIV, gastritis, thalassemia, G6PD, anemia, DM, HTN, dyslipidemia, lung disease, cancer, autoimmunity, kidney disease, and rheumatism (Table 2 and see Table S1 in the Additional file).

**Table 2:** The sign and symptoms, laboratory factors, comorbidities and vital signs in a COVID-19 patient cohort

Variable	Levels	Total (*n* = 442)	Outcome		*P*-value
			**Discharge (*n* = 351)**	**Deceased (*n* = 34)**	
**Sign and Symptoms**					
Temperature	–––	36.41 ± 5.81	36.72 ± 4.59	38.20 ± 0.84	0.009^a^
Halitosis	No	199 (45.02)	159 (45.30)	9 (26.47)	0.043^c^
	Yes	225 (50.90)	180 (51.28)	23 (67.65)	
**Laboratory Findings**					
TPR	–––	37 (36.9,37.7)	37 (36.8,37.6)	37.2 (37,38.2)	< 0.001^b^
PR	–––	89 (80,104)	89 (80,104)	90 (82,114)	0.001^b^
RR	–––	19 (18,20)	19 (18,20)	20 (18,23)	< 0.001^b^
BP	–––	129 (110,140)	130 (110,140)	120 (105,142)	< 0.001^b^
SPO2	–––	90 (85,94)	91 (85,94)	83.5 (76,90)	< 0.001^b^
PO2	–––	35 (27,49)	36 (27,49)	30.5 (27.25,45)	0.001^b^
GCS	–––	15 (14,15)	15 (14,15)	13.5 (10.5,15)	0.004^b^
WBC	–––	7.7 (5.2,10.6)	7.7 (5.2,10.55)	9.14 (5,11.975)	< 0.001^b^
HB	–––	13.2 (11.85,14.6)	13.15 (11.875,14.725)	13 (11.2,14.3)	< 0.001^b^
PLT	–––	204 (153,254)	210 (155,260)	173 (133.1,237)	< 0.001^b^
EOSINO	–––	1 (0.1,2)	0.9 (0.1,2)	1.15 (0.275,2)	0.001^b^
BASO	–––	0.3 (0.2,1)	0.3 (0.2,0.925)	0.2 (0.1,2)	0.001^b^
NUT	–––	72.4 (60,81.8)	72.4 (60,81.7)	79 (69.05,86.875)	< 0.001^b^
LYMP	–––	18 (12,26.075)	18 (12,27)	14.1 (10,20.625)	< 0.001^b^
ESR	–––	43 (26,66)	41.4 (27.75,65.25)	35 (17,56.5)	< 0.001^b^
CRP	–––	20.14 (2,72.2425)	16.12 (1.95,68.115)	43 (2.75,90.67)	< 0.001^b^
PT	–––	13.3 (13,15)	13 (13,15)	14 (12.5,15.825)	0.001^b^
PTT	–––	33 (29.825,37)	33 (30,37.7)	33 (29.3,35.125)	0.001^b^
INR	–––	1.09 (1,1.28)	1.035 (1,1.21)	1.1 (1.02,1.3375)	< 0.001^b^
VITD	–––	34.48 (19.03,44.99)	35.72 (19.03,44.99)	56.71 (31.82,75.64)	0.021^b^
FR	–––	297.9 (171.4,519.5)	300.3 (186.0,548.1)	NA (NA,NA)	––
FBS	–––	129 (107,155)	128 (107.5,159.5)	141 (99,195)	0.001^b^
BUN	–––	24 (16.395,45.5)	23.4 (15.9,41)	40.5 (25.4,65.75)	< 0.001^b^
CR	–––	1.15 (0.9725,1.5)	1.15 (0.97,1.48)	1.3 (1,2.05)	< 0.001^b^
NA	–––	136 (133,139)	136 (133,139)	135 (132.25,138.75)	< 0.001^b^
K	–––	4.3 (3.9,4.6)	4.3 (3.9,4.6)	4.125 (3.67,4.79)	< 0.001^b^
CA	–––	8.7 (8.1,9.2)	8.7 (8.1,9.2)	8.8 (8.65,9.2)	0.002^b^
LDH	–––	485.5 (341.1,671)	483 (333.7,660.5)	730.5 (645,997)	< 0.001^b^
LDL	–––	71.4 (51.25,93)	73 (53,96)	75.5 (71.75,79.25)	0.008^b^
TG	–––	104 (87,140)	100.5 (86.25,138.5)	124.5 (96,188.25)	0.003^b^
HDL	–––	34 (24,44.25)	35 (26,45)	21.5 (20.25,22.75)	0.005^b^
AST	–––	37 (27,55)	37 (26.5,54)	64 (40.25,128.5)	< 0.001^b^
ALT	–––	30 (18,44.25)	30 (18,44.5)	39 (35.5,55.5)	< 0.001^b^

Quantitative variables are presented as mean ± standard deviation (SD) or median (interquartile range [IQR]). Qualitative variables are reported as frequency (percentage). a) An independent t-test was utilized to compare the means of quantitative variables with an approximately normal distribution between the discharged and deceased groups. b) The exact Mann–Whitney U test was employed to compare the non-normal distribution of quantitative data between the discharged and deceased groups. c) Fisher's exact test was applied to assess the association between qualitative variables and the outcome variable

#### Balance checking the PSM for ICU admission, mortality, and need for intubation

**Fig. 2:**
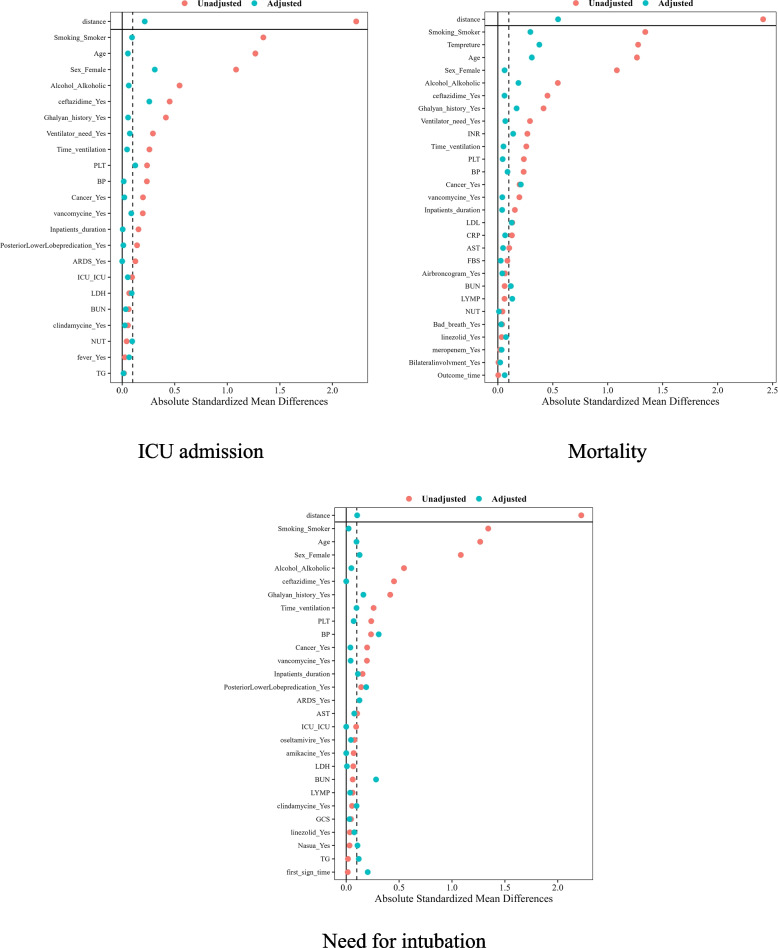
Absolute standardized mean difference for evaluating covariate balance between treated and non-treated groups in relation to ICU admission, mortality, or need for intubation

In the subsequent phase, the objective is to mitigate the influence of confounding variables on the outcomes and focus solely on assessing the impact of Opium. To achieve this, the Propensity Score Matching (PSM) model was implemented, effectively attenuating the confounding effects for ICU admission, mortality, and the need for intubation, as demonstrated in Fig. 2. The results presented in Fig. 2 provide empirical evidence supporting the effectiveness of the PSM model in achieving balance among medication- or ICU-related confounding variables. Out of the initial set of 22 variables, 19 exhibit a significant level of balance between the two drug groups, confirming the successful balancing accomplished by the PSM model. Furthermore, Fig. 2 illustrates that, among the 38 confounding variables examined, 28 variables have reached a state of equilibrium and balance between the two treatment groups. As a result, the impact of these variables on mortality has been effectively mitigated. Additionally, upon analysis of Fig. 2, it is evident that 17 out of the 27 confounding variables assessed have achieved balance and equilibrium between the two treatment groups, effectively eliminating their influence on the need for intubation. It is important to note that a detailed assessment of Table S2, S3, and S4 highlighted the insignificance of differences in covariates between the non-user opium and user opium groups post the implementation of the PSM approach (see Additional file 1).

#### The impact of opium use on ICU hospitalization rates among COVID-19 patients

The impact of opium use on ICU rates, mortality, and the need for intubation among hospitalized COVID-19 patients was examined. Table 3 presents the results obtained from different models and analyses.

**Table 3:** The impact of opium use on the ICU rates, mortality, and need for intubation among hospitalized COVID-19 patients

Treatment	Outcome	Model	OR (95% CI)	*P*-value
Opium	ICU admission	PSM	1.87 (0.22, 2.91)	0.631
		Unadjusted	1.76 (0.36, 2.59)	0.467
		Full adjusted	–––	––
		Optimal model	1.42 (0.14, 2.24)	0.116
Opium	Mortality	PSM	2.38 (1.30–4.35)	0.005
		Unadjusted	2.19 (0.52, 3.74)	0.679
		Full adjusted	2.34 (0.17, 3.74)	0.697
		Optimal model	2.39 (0.13, 3.19)	0.571
Opium	Need for intubation	PSM	3.57 (1.38, 9.39)	0.009
		Unadjusted	2.01 (0.97, 4.17)	0.062
		Full adjusted	5.54 (1.97, 15.61)	0.001
		Optimal model	6.34 (2.59, 15.50)	< 0.001

A full adjustment method was implemented, considering all variables. The selection of the optimal model was carried out through a backward stepwise algorithm. In the propensity score matching (PSM) approach, variables exhibiting significant associations with outcomes (ICU admission or mortality or need for intubation) or opioid use were taken into consideration. Caliper parameters were set at 0.5 for the three response variables, while the ratio was established at 3, 3, and 4 correspondingly. Potential confounding variables were carefully matched between the group of individuals who received opioids and the group of individuals who did not receive opioids

Regarding ICU admission, the propensity score matching (PSM) approach showed that opium use was not significantly associated with ICU admission (OR: 1.87, 95% CI: 0.22–2.91, *p* = 0.631). Similar findings were observed in the unadjusted model (OR: 1.76, 95% CI: 0.36–2.59, *p* = 0.467).

#### The impact of opium use on mortality among hospitalization COVID-19 patients

In terms of mortality, opium use exhibited a significant association in the PSM model (OR = 2.38 95% CI: 1.30–4.35, *p* = 0.005) indicating an increased risk of mortality among opium users. However, this association was not observed in the unadjusted model (OR: 2.19, 95% CI: 0.52–3.74, *p* = 0.679) or the fully adjusted model (OR: 2.34, 95% CI: 0.17–3.74, *p* = 0.69). The optimal model yielded a similar result, indicating a non-significant effect of opium use (OR: 2.39, 95% CI: 0.13–3.19, *p* = 0.57).

#### The impact of opium use on the need for intubation in COVID-19 patients

Regarding the need for intubation, opium use showed a significant association in all models. In the PSM approach, opium users had a higher likelihood of requiring intubation (OR: 3.57, 95% CI: 1.38–9.39, *p* = 0.009). This association was also observed in the unadjusted model (OR: 2.01, 95% CI: 0.97–4.17, *p* = 0.062) and the fully adjusted model (OR: 5.54, 95% CI: 1.97–15.61, *p* = 0.001). The optimal model yielded the highest odds ratio, suggesting a strong association between opium use and the need for intubation (OR: 6.34, 95% CI: 2.59–15.50, *p* < 0.001).

#### The impact of opium use on the severity of pulmonary involvement in CT scans of hospitalized COVID-19 patients

The study investigated the impact of opium use on the severity of pulmonary involvement in CT scans of hospitalized COVID-19 patients. Table 4 presents the results obtained from various models and analyses.

**Table 4:** The Impact of Opium Use on the Severity of Pulmonary Involvement in CT scans of Hospitalized COVID-19 Patients

Outcomes	Model	OR (95% CI)	*P*-value
Ground Glass Opacities	Unadjusted	1.26 (0.50, 3.21)	0.627
	Full adjusted	0.66 (0.09, 4.62)	0.672
	Optimal model	0.76 (0.14, 4.18)	0.752
Posterior Lower Lobe Prediction	Unadjusted	0.75 (0.32, 1.77)	0.511
	Full adjusted	0.64 (0.24, 1.71)	0.374
	Optimal model	0.66 (0.37, 1.18)	0.163
Pure Consolidation	Unadjusted	0.74 (0.23, 2.40)	0.617
	Full adjusted	3.56 (0.29, 44.02)	0.323
	Optimal model	1.97 (0.62, 6.26)	0.251
Peripheral Subpleural distribution	Unadjusted	0.49 (0.21, 1.17)	0.108
	Full adjusted	1.31 (0.40, 4.24)	0.655
	Optimal model	1.34 (0.45, 3.94)	0.598
Pleural Effusion	Unadjusted	0.49 (0.06, 4.20)	0.519
	Full adjusted	–––	–––
	Optimal model	–––	–––
Multiple Lesions	Unadjusted	0.99 (0.42, 2.33)	0.980
	Full adjusted	0.66 (0.23, 1.86)	0.429
	Optimal model	0.69 (0.26, 1.81)	0.453
Crazy Paving Pattern	Unadjusted	2.35 (0.77, 7.15)	0.134
	Full adjusted	–––	–––
	Optimal model	5.45 (0.44, 474.33)	0.118
Air Bronco Gram	Unadjusted	0.33 (0.07, 1.52)	0.154
	Full adjusted	–––	–––
	Optimal model	–––	–––
Bilateral Involvement	Unadjusted	0.97 (0.43, 2.16)	0.935
	Full adjusted	0.89 (0.30, 2.68)	0.836
	Optimal model	0.90 (0.30, 2.66)	0.483

A full adjustment method was employed, incorporating all variables, and the optimal model was determined using a stepwise backward algorithm

For all outcomes assessed, including ground glass opacities, posterior lower lobe prediction, pure consolidation, peripheral subpleural distribution, pleural effusion, multiple lesions, crazy paving pattern, air bronchogram, and bilateral involvement, opium use did not show a significant association. These findings were consistent across different models, including unadjusted, fully adjusted, and optimal models.

## Discussion

In this study, we investigated the potential link between opioid usage and clinical outcomes in COVID-19 patients. We employed the propensity score matching (PSM) method to analyze the data. Our comprehensive power analysis demonstrated that the logistic regression models, focusing on the effect of opium use, were well-powered, even with the reduced sample size post-matching. Initially, our unadjusted analyses did not find any significant association between opioid usage and outcomes such as ICU admission, mortality, and the need for mechanical ventilation. However, upon adjusting for covariates, the results of the PSM analysis revealed a significant association between opioid use and increased mortality rate as well as a higher likelihood of requiring mechanical ventilation.

It is important to acknowledge the limitations of our study. One limitation of our study is the relatively small sample size. Although we employed propensity score matching to minimize confounding factors, the limited number of participants may have impacted the statistical power of our analysis and introduced potential bias. Also, the study was conducted in a specific setting and may not be fully representative of the general population. The findings may not be applicable to other regions or populations with different demographic characteristics, healthcare systems, or prevalence of opioid use disorder. Our study relied on retrospective data collection, which is subject to inherent limitations such as recall bias and incomplete or inaccurate medical records. Additionally, the observational nature of the study design prevents us from establishing causal relationships between opioid use disorder and COVID-19 outcomes.

The COVID-19 pandemic has drawn attention to the potential impact of comorbidities, particularly substance use disorders (SUDs), on the prognosis of the disease [25]. It is essential to comprehend the effects of SUDs, including opioids, on COVID-19 outcomes to guide clinical management and implement effective public health interventions [26]. Substance use disorder has been shown to weaken the immune system, increase the risk of respiratory complications, and contribute to overall health deterioration, all of which may influence the course and severity of COVID-19 [25]. Opioids also possess immunomodulatory properties that may influence the immune response to SARS-CoV-2, the virus responsible for COVID-19 [27].

Several studies have investigated the association between opioid use and COVID-19 outcomes, including disease severity, hospitalization rates, intensive care unit (ICU) admissions, and mortality [28–34]. However, it is important to note that the evidence in this area is limited, and the available studies have produced conflicting results. Two recent meta-analyses have conducted a quantitative pooling of available data on the relationship between opioid use and COVID-19 outcomes [14, 35]. In the study by Ao et al. [14], it was found that opioid use disorder significantly increased the rate of mortality and ICU admission in COVID-19 patients. However, they mentioned that the need for mechanical ventilation did not show a significant difference between the two groups, which contrasts with our findings. It is important to acknowledge that this finding was based on limited evidence, as only two studies were included in their analysis. Conversely, our study strengthens the evidence regarding the detrimental impact of opioid use on the need for mechanical ventilation, as the individual results of each study included in the mentioned meta-analysis showed a significant increase in this requirement [32, 33]. The most recent meta-analysis conducted by Behnoush et al. yielded similar findings to the earlier meta-analysis [35]. They also confirmed that the opioid group had a significantly higher rate of hospitalization. Additionally, they examined whether prior exposure to opioids affected the rate of COVID-19 acquisition, but their findings did not support an increased rate of COVID-19 among these individuals. It is important to note that both of these meta-analyses are limited by the inclusion of only five studies, leading to a low certainty of evidence regarding their outcomes.

Our findings are generally consistent with the existing literature, which suggests an overall elevated risk of severe COVID-19 among individuals with opioid addiction [3, 14, 30, 32–35]. However, it is important to acknowledge studies that have reported non-significant findings between the opioid and non-opioid groups. Our study did not show any relation between opioid usage and increased rate of ICU admission, which is in contrast with what previous studies have found [3, 14]. Furthermore, when utilizing the PSM method for our univariate analysis of mortality rates, we observed a significant increase in the mortality rate among the opioid group. This contrasts with the findings of Qaeden [32] and Wiener [34], who reported a significant association in their univariate analysis but a non-significant association after adjusting for confounding variables. The study conducted by Allen et al. did not identify any association with mortality rates in both unadjusted and adjusted analyses [29].

One potential explanation for the conflicting findings could be the presence of confounding factors. Individuals who are known as opioid users may have underlying health conditions or comorbidities such as cardiovascular disease, diabetes, and liver disease that increase their susceptibility to severe COVID-19 outcomes [36]. The synergistic effect of SUDs and chronic health conditions can further weaken the immune system, exacerbate inflammation, and impair overall organ function, increasing the likelihood of severe disease progression [37]. In terms of clinical management, the use of opioids in COVID-19 patients poses challenges and considerations [38]. Opioids can cause respiratory depression, which is a significant concern in individuals with COVID-19-related respiratory distress [39].

Nevertheless, the immunomodulatory properties of opioids raise concerns about their potential impact on the immune response to SARS-CoV-2 [40]. Opioids have been shown to suppress immune responses, including the activity of natural killer cells, T cells, and macrophages, which play vital roles in clearing viral infections [41]. By impairing the immune response, opioids may contribute to prolonged viral replication and increased disease severity in COVID-19 patients [27]. Additionally, opioids can alter cytokine production, leading to dysregulated inflammation, which is a hallmark of severe COVID-19 [40]. The effects of opioids on the immune response to SARS-CoV-2 have been explored in a limited number of studies. Studies showed that opioid use was associated with lower levels of pro-inflammatory cytokines in COVID-19 patients, suggesting a potential blunting of the immune response [42]. In addition, evidence indicates that opium can escalate the rate of mortality by reducing the of IFNs expression, developing pulmonary edema, increasing the expression of Angiotensin-converting enzyme 2(ACE2) and escalating the thrombotic factors level [43]. However, more research is needed to elucidate the specific mechanisms by which opioids interact with the immune system and how these interactions impact COVID-19 outcomes.

Addressing the opioid use disorder impacts on COVID-19 patients requires comprehensive public health interventions [4]. Educational campaigns aimed at healthcare providers and patients can raise awareness about the risks of opioids in the context of COVID-19 [30]. Improved access to addiction treatment services is essential for individuals struggling with opioid use disorders during these challenging times [44].

In addition to the clinical implications, investigating the effect of opioid consumption on COVID-19 prognosis has broader public health implications [45]. The COVID-19 pandemic has highlighted the importance of addressing the opioid epidemic and reducing unnecessary opioid prescriptions [46]. By understanding the potential impact of opioids on COVID-19 outcomes, public health interventions can be designed to minimize the risks associated with opioid [3].

Moreover, harm reduction approaches can promote safer substance use practices, reducing the risk of respiratory complications and infections [47].

While the existing evidence is limited and inconclusive, further research is needed to better understand the relationship between opioid consumption and COVID-19 prognosis. Prospective studies and randomized controlled trials are warranted to establish a causal link between opioids and COVID-19 outcomes including hospitalization, mortality, intubation, ICU admission and O2 saturation. These studies should include larger sample sizes and consider potential confounding factors, such as underlying health conditions and comorbidities.

Future research should also explore the impact of different opioid types, dosages, and durations of use on COVID-19 outcomes. It is possible that specific opioids or certain patterns of opioid use may have varying effects on the immune response and disease severity.

In conclusion, our study provides compelling evidence that opium users are at a significantly higher risk of experiencing severe COVID-19 outcomes, including mortality and the need for mechanical ventilation. These findings underscore the importance of recognizing the impact of opium use on COVID-19 prognosis and the need for tailored interventions for this vulnerable population. Further research in this area is warranted to deepen our understanding, guide clinical decision-making, improve patient outcomes, and inform public health policies aimed at mitigating the adverse effects of opium use on COVID-19 outcomes.

### Supplementary Information

**Additional file 1: Table S1.** The sign and symptoms, laboratory factors, comorbidities and vital signs in a COVID-19 patient cohort.

## Data Availability

The dataset used and analyzed during the current study available from the corresponding author on reasonable request.
