# Supplementary material for: Association between opioid abuse and COVID-19 susceptibility: a propensity score matched study
Source: BMC Infect Dis. 2023 Dec 5;23:851. doi: 10.1186/s12879-023-08842-4 (PMC10696756; doi:10.1186/s12879-023-08842-4)
Supplement: Supplementary file 1 — Additional file 1: Table S1. The sign and symptoms, laboratory factors, comorbidities and vital signs in a COVID-19 patient cohort. [file 12879_2023_8842_MOESM1_ESM.docx]

Table S1. The Sign and Symptoms, Laboratory Factors, Comorbidities and Vital Signs in a COVID-19 Patient Cohort

| **Variable** | **Levels** | **Total (n=442)** | **Outcome** | | | **P-value** |
| --- | --- | --- | --- | --- | --- | --- |
|  |  |  | **Discharge (n=351)** | | **Deceased (n=34)** |  |
| **Sign and Symptoms** |  |  |  | |  |  |
| Temperature | ----- | 36.41 ± 5.81 | 36.72 ± 4.59 | | 38.20 ± 0.84 | 0.009^a^ |
| The time of onset of the first symptom | ----- | 6 (3 ,9) | 6 (2 ,9) | | 7 (3.5 ,10) | 0.174^b^ |
| Number of individuals exposed to contact | ----- | 4 (2 ,5) | 4 (2 ,5) | | 4 (2 ,5.25) | 0.779^b^ |
| Time interval from the first symptom to admission | ----- | 3 (2 ,7) | 3 (2 ,7) | | 2 (2 ,3.75) | 0.239^b^ |
| Fever | No | 243 (54.98) | 189 (53.85) | | 17 (50.00) | 0.853^c^ |
|  | Yes | 179 (40.50) | 148 (42.17) | | 15 (44.12) |  |
| Caught | No | 214 (48.42) | 162 (46.15) | | 20 (58.82) | 0.139 ^c^ |
|  | Yes | 209 (47.29) | 176 (50.14) | | 12 (35.29) |  |
| Septum | No | 395 (89.37) | 313 (89.17) | | 31 (91.18) | 0.380 ^c^ |
|  | Yes | 19 (4.30) | 17 (4.84) | | 0 (0.00) |  |
| Halitosis | No | 199 (45.02) | 159 (45.30) | | 9 (26.47) | 0.043 ^c^ |
|  | Yes | 225 (50.90) | 180 (51.28) | | 23 (67.65) |  |
| Sore throat | No | 405 (91.63) | 322 (91.74) | | 32 (94.12) | 0.380 ^c^ |
|  | Yes | 18 (4.07) | 16 (4.56) | | 0 (0.00) |  |
| Rhinorrhea | No | 415 (93.89) | 332 (94.59) | | 32 (94.12) | 1.000 ^c^ |
|  | Yes | 8 (1.81) | 6 (1.71) | | 0 (0.00) |  |
| Headache | No | 379 (85.75) | 303 (86.32) | | 31 (91.18) | 0.759 ^c^ |
|  | Yes | 45 (10.18) | 35 (9.97) | | 2 (5.88) |  |
| Muscle pain | No | 333 (75.34) | 267 (76.07) | | 26 (76.47) | 1.000 ^c^ |
|  | Yes | 90 (20.36) | 71 (20.23) | | 6 (17.65) |  |
| Loss of appetite | No | 313 (70.81) | 249 (70.94) | | 24 (70.59) | 1.000 ^c^ |
|  | Yes | 109 (24.66) | 88 (25.07) | | 8 (23.53) |  |
| Diarrhea | No | 415 (93.89) | 331 (94.30) | | 32 (94.12) | 1.000 ^c^ |
|  | Yes | 7 (1.58) | 6 (1.71) | | 0 (0.00) |  |
| Nausea | No | 383 (86.65) | 309 (88.03) | | 31 (91.18) | 0.493 ^c^ |
|  | Yes | 39 (8.82) | 28 (7.98) | | 1 (2.94) |  |
| Vomiting | No | 396 (89.59) | 315 (89.74) | | 31 (91.18) | 0.708 ^c^ |
|  | Yes | 26 (5.88) | 22 (6.27) | | 1 (2.94) |  |
| Dizziness | No | 409 (92.53) | 328 (93.45) | | 31 (91.18) | 0.601 ^c^ |
|  | Yes | 13 (2.94) | 9 (2.56) | | 1 (2.94) |  |
| **Laboratory Findings** |  |  |  | |  |  |
| TPR | ----- | 37 (36.9 ,37.7) | 37 (36.8 ,37.6) | | 37.2 (37 ,38.2) | <0.001 ^b^ |
| PR | ----- | 89 (80 ,104) | 89 (80 ,104) | | 90 (82 ,114) | 0.001 ^b^ |
| RR | ----- | 19 (18 ,20) | 19 (18 ,20) | | 20 (18 ,23) | <0.001 ^b^ |
| BP | ----- | 129 (110 ,140) | 130 (110 ,140) | | 120 (105 ,142) | <0.001 ^b^ |
| SPO2 | ----- | 90 (85 ,94) | 91 (85 ,94) | | 83.5 (76 ,90) | <0.001 ^b^ |
| PO2 | ----- | 35 (27 ,49) | 36 (27 ,49) | | 30.5 (27.25 ,45) | 0.001 ^b^ |
| GCS | ----- | 15 (14 ,15) | 15 (14 ,15) | | 13.5 (10.5 ,15) | 0.004 ^b^ |
| WBC | ----- | 7.7 (5.2 ,10.6) | 7.7 (5.2 ,10.55) | | 9.14 (5 ,11.975) | <0.001 ^b^ |
| HB | ----- | 13.2 (11.85 ,14.6) | 13.15 (11.875 ,14.725) | | 13 (11.2 ,14.3) | <0.001 ^b^ |
| PLT | ----- | 204 (153 ,254) | 210 (155 ,260) | | 173 (133.1 ,237) | <0.001 ^b^ |
| EOSINO | ----- | 1 (0.1 ,2) | 0.9 (0.1 ,2) | | 1.15 (0.275 ,2) | 0.001 ^b^ |
| BASO | ----- | 0.3 (0.2 ,1) | 0.3 (0.2 ,0.925) | | 0.2 (0.1 ,2) | 0.001 ^b^ |
| NUT | ----- | 72.4 (60 ,81.8) | 72.4 (60 ,81.7) | | 79 (69.05 ,86.875) | <0.001 ^b^ |
| LYMP | ----- | 18 (12 ,26.075) | 18 (12 ,27) | | 14.1 (10 ,20.625) | <0.001 ^b^ |
| ESR | ----- | 43 (26 ,66) | 41.4 (27.75 ,65.25) | | 35 (17 ,56.5) | <0.001 ^b^ |
| CRP | ----- | 20.14 (2 ,72.2425) | 16.12 (1.95 ,68.115) | | 43 (2.75 ,90.67) | <0.001 ^b^ |
| PT | ----- | 13.3 (13 ,15) | 13 (13 ,15) | | 14 (12.5 ,15.825) | 0.001 ^b^ |
| PTT | ----- | 33 (29.825 ,37) | 33 (30 ,37.7) | | 33 (29.3 ,35.125) | 0.001 ^b^ |
| INR | ----- | 1.09 (1 ,1.28) | 1.035 (1 ,1.21) | | 1.1 (1.02 ,1.3375) | <0.001 ^b^ |
| VITD | ----- | 34.48 (19.03 ,44.99) | 35.72 (19.03 ,44.99) | | 56.71 (31.82 ,75.64) | 0.021 ^b^ |
| FR | ----- | 297.9 (171.4 ,519.5) | 300.3 (186.0 ,548.1) | | NA (NA ,NA) | ---- |
| FBS | ----- | 129 (107 ,155) | 128 (107.5 ,159.5) | | 141 (99 ,195) | 0.001 ^b^ |
| BUN | ----- | 24 (16.395 ,45.5) | 23.4 (15.9 ,41) | | 40.5 (25.4 ,65.75) | <0.001 ^b^ |
| CR | ----- | 1.15 (0.9725 ,1.5) | 1.15 (0.97 ,1.48) | | 1.3 (1 ,2.05) | <0.001 ^b^ |
| NA. | ----- | 136 (133 ,139) | 136 (133 ,139) | | 135 (132.25 ,138.75) | <0.001 ^b^ |
| K | ----- | 4.3 (3.9 ,4.6) | 4.3 (3.9 ,4.6) | | 4.125 (3.67 ,4.79) | <0.001 ^b^ |
| CA | ----- | 8.7 (8.1 ,9.2) | 8.7 (8.1 ,9.2) | | 8.8 (8.65 ,9.2) | 0.002 ^b^ |
| LDH | ----- | 485.5 (341.1 ,671) | 483 (333.7 ,660.5) | | 730.5 (645 ,997) | <0.001 ^b^ |
| LDL | ----- | 71.4 (51.25 ,93) | 73 (53 ,96) | | 75.5 (71.75 ,79.25) | 0.008 ^b^ |
| TG | ----- | 104 (87 ,140) | 100.5 (86.25 ,138.5) | | 124.5 (96 ,188.25) | 0.003 ^b^ |
| HDL | ----- | 34 (24 ,44.25) | 35 (26 ,45) | | 21.5 (20.25 ,22.75) | 0.005 ^b^ |
| AST | ----- | 37 (27 ,55) | 37 (26.5 ,54) | | 64 (40.25 ,128.5) | <0.001 ^b^ |
| ALT | ----- | 30 (18 ,44.25) | 30 (18 ,44.5) | | 39 (35.5 ,55.5) | <0.001 ^b^ |
| DDIMER | ----- | 200 (0.075 ,375) | 200 (0.75 ,375) | | 300 (150 ,450) | 0.224 ^b^ |
| **Comorbidities** |  |  |  |  | |  |
| Pregnancy | No | 294 (66.52) | 235 (66.95) | 22 (64.71) | | ---- |
|  | Yes | 0 (0.00) | 0 (0.00) | 0 (0.00) | |  |
| HBB | No | 407 (92.08) | 322 (91.74) | (94.12) 32 | | 1.000 ^c^ |
|  | Yes | 1 (0.23) | 1 (0.28) | (0.00) 0 | |  |
| HBc | No | 407 (92.08) | 322 (91.74) | (94.12) 32 | | 1.000 ^c^ |
|  | Yes | 1 (0.23) | 1 (0.28) | (0.00) 0 | |  |
| HIV | No | 408 (92.31) | 323 (92.02) | (94.12) 32 | | ---- |
|  | Yes | 0 (0.00) | 0 (0.00) | (0.00) 0 | |  |
| Gastit | No | 382 (86.43) | 301 (85.75) | (85.29) 29 | | 1.000 ^c^ |
|  | Yes | 2 (0.45) | 2 (0.57) | (0.00) 0 | |  |
| Thalassemia | No | 405 (91.63) | 320 (91.17) | (94.12) 32 | | 1.000 ^c^ |
|  | Yes | 1 (0.23) | 1 (0.28) | (0.00) 0 | |  |
| G6PD | No | 402 (90.95) | 318 (90.60) | (94.12) 32 | | 1.000 ^c^ |
|  | Yes | 2 (0.45) | 2 (0.57) | (0.00) 0 | |  |
| Anemia | No | 399 (90.27) | 314 (89.46) | (94.12) 32 | | 1.000 ^c^ |
|  | Yes | 6 (1.36) | 6 (1.71) | (0.00) 0 | |  |
| CVD | No | 291 (65.84) | 232 (66.10) | (55.88) 19 | | 0.423 ^c^ |
|  | Yes | 137 (31.00) | 112 (31.91) | (38.24) 13 | |  |
| DM | No | 322 (72.85) | 254 (72.36) | (73.53) 25 | | 0.839 ^c^ |
|  | Yes | 109 (24.66) | 92 (26.21) | (23.53) 8 | |  |
| HTN | No | 267 (60.41) | 218 (62.11) | (50.00) 17 | | 0.340 ^c^ |
|  | Yes | 163 (36.88) | 127 (36.18) | (44.12) 15 | |  |
| Dyslipidemia | No | 380 (85.97) | 302 (86.04) | (88.24) 30 | | 0.229 ^c^ |
|  | Yes | 42 (9.50) | 37 (10.54) | (2.94) 1 | |  |
| Lung disease | No | 353 (79.86) | 286 (81.48) | (73.53) 25 | | 1.000 ^c^ |
|  | Yes | 76 (17.19) | 60 (17.09) | (20.59) 7 | |  |
| Cancer | No | 413 (93.44) | 332 (94.59) | (91.18) 31 | | 1.000 ^c^ |
|  | Yes | 16 (3.62) | 13 (3.70) | (2.94) 1 | |  |
| Autoimmunity | No | 423 (95.70) | 340 (96.87) | (91.18) 31 | | 0.415 ^c^ |
|  | Yes | 6 (1.36) | 5 (1.42) | (2.94) 1 | |  |
| Kidney disease | No | 406 (91.86) | 329 (93.73) | (85.29) 29 | | 0.212 ^c^ |
|  | Yes | 23 (5.20) | 16 (4.56) | (8.82) 3 | |  |
| Rheumatism | No | 419 (94.80) | 336 (95.73) | (94.12) 32 | | 1.000 ^c^ |
|  | Yes | 9 (2.04) | 8 (2.28) | (0.00) 0 | |  |
| Thyroid | No | 404 (91.40) | 324 (92.31) | (88.24) 30 | | 1.000 ^c^ |
|  | Yes | 18 (4.07) | 15 (4.27) | (2.94) 1 | |  |
| Liver dysfunction | No | 337 (76.24) | 271 (77.21) | 29 (85.29) | | 1.000 ^c^ |
|  | Yes | 2 (0.45) | 2 (0.57) | 0 (0.00) | |  |
| Coagulation disorder | No | 335 (75.79) | 269 (76.64) | 29 (85.29) | | 1.000 ^c^ |
|  | Yes | 3 (0.68) | 3 (0.85) | 0 (0.00) | |  |

Note: Quantitative variables are presented as mean ± standard deviation (SD) or median (interquartile range [IQR]). Qualitative variables are reported as frequency (percentage). a) An independent t-test was utilized to compare the means of quantitative variables with an approximately normal distribution between the discharged and deceased groups. b) The exact Mann-Whitney U test was employed to compare the non-normal distribution of quantitative data between the discharged and deceased groups. c) Fisher's exact test was applied to assess the association between qualitative variables and the outcome variable.

**Table S2**. Patients’ characteristics, comorbidities, treatments, and laboratory tests before and after propensity score matching [For outcome = ICU]

| **Variable** | **Level** | **Before Propensity Score-Matched patients** | | | **After Propensity Score-Matched patients** | | |
| --- | --- | --- | --- | --- | --- | --- | --- |
|  |  | **Non-user Opium (n=337)** | **User Opium (n=105)** | **P-value** | **Non-user Opium (n=65)** | **User Opium (n=31)** | **P-value** |
| Age | ----- | 80.48 ± 5.70 | 59.59 ± 16.49 | <0.001 | 77.53 ± 4.18 | 76.65 ± 8.34 | 0.852 |
| BP | ----- | 128.62 ± 22.26 | 123.51 ± 21.74 | 0.024 | 121.71 ± 20.46 | 121.81 ± 27.61 | 0.694 |
| PLT | ----- | 212.09 ± 90.08 | 237.10 ± 105.67 | 0.018 | 233.82 ± 110.19 | 220.65 ± 61.26 | 0.905 |
| NUT | ----- | 67.23 ± 22.17 | 66.18 ± 25.10 | 0.801 | 68.10 ± 24.06 | 66.22 ± 27.82 | 0.957 |
| BUN | ----- | 37.90 ± 43.63 | 35.24 ± 43.20 | 0.079 | 47.49 ± 56.09 | 49.35 ± 52.73 | 0.617 |
| LDH | ----- | 564.03 ± 364.59 | 541.86 ± 332.60 | 0.166 | 614.14 ± 580.36 | 592.06 ± 376.00 | 0.823 |
| TG | ----- | 117.45 ± 56.69 | 118.30 ± 50.85 | 0.464 | 128.59 ± 72.29 | 129.35 ± 56.75 | 0.588 |
| Time_ventilation | ----- | 0.72 ± 1.82 | 1.38 ± 2.57 | 0.010 | 1.18 ± 2.01 | 1.06 ± 2.13 | 0.443 |
| Inpatients_duration | ----- | 6.03 ± 4.93 | 5.32 ± 4.54 | 0.176 | 5.35 ± 3.72 | 5.29 ± 3.40 | 0.868 |
| Sex | Male | 175 (0.52) | 92 (87.62) | <0.001 | 44 (67.20) | 24 (77.42) | 0.472 |
| Sex | Female | 162 (0.48) | 13 (12.38) |  | 21 (32.80) | 7 (22.58) |  |
| Smoking | Non-smoker | 311 (0.92) | 32 (30.48) | <0.001 | 41 (63.44) | 21 (67.74) | 0.827 |
| Smoking | Smoker | 26 (0.08) | 73 (69.52) |  | 24 (36.56) | 10 (32.26) |  |
| Alcohol | Non-alkoholic | 333 (0.99) | 79 (75.24) | <0.001 | 61 (94.09) | 30 (96.77) | 1.000 |
| Alcohol | Alkoholic | 4 (0.01) | 26 (24.76) |  | 4 (5.91) | 1 (3.23) |  |
| opium | Non-user | 337 (1.00) | 0 (0.00) |  | 65 (100.00) | 0 (0.00) |  |
| opium | User | 0 (0.00) | 105 (100.00) |  | 37 (56.99) | 31 (100.00) |  |
| fever | No | 195 (0.58) | 62 (59.05) | 0.919 | 28 (43.01) | 19 (61.29) | 0.854 |
| fever | Yes | 142 (0.42) | 43 (40.95) |  | 63 (97.31) | 12 (38.71) |  |
| Cancer | No | 329 (0.98) | 97 (92.38) | 0.027 | 2 (2.69) | 30 (96.77) | 1.000 |
| Cancer | Yes | 8 (0.02) | 8 (7.62) |  | 60 (92.47) | 1 (3.23) |  |
| Ghalyan_history | No | 330 (0.98) | 86 (81.90) | <0.001 | 5 (7.53) | 28 (90.32) | 0.710 |
| Ghalyan_history | Yes | 7 (0.02) | 19 (18.10) |  | 59 (90.32) | 3 (9.68) |  |
| ARDS | No | 304 (0.90) | 98 (93.33) | 0.435 | 6 (9.68) | 28 (90.32) | 1.000 |
| ARDS | Yes | 33 (0.10) | 7 (6.67) |  | 38 (58.06) | 3 (9.68) |  |
| Ventilator_need | No | 288 (0.85) | 76 (72.38) | 0.003 | 27 (41.94) | 17 (54.84) | 0.909 |
| Ventilator_need | Yes | 49 (0.15) | 29 (27.62) |  | 28 (42.47) | 14 (45.16) |  |
| PosteriorLowerLobepredication | No | 124 (0.37) | 46 (43.81) | 0.240 | 37 (57.53) | 13 (41.94) | 1.000 |
| PosteriorLowerLobepredication | Yes | 213 (0.63) | 59 (56.19) |  | 61 (93.55) | 18 (58.06) |  |
| ceftazidime | No | 302 (0.90) | 102 (97.14) | 0.028 | 4 (6.45) | 30 (96.77) | 1.000 |
| ceftazidime | Yes | 35 (0.10) | 3 (2.86) |  | 59 (90.86) | 1 (3.23) |  |
| clindamycine | No | 317 (0.94) | 100 (95.24) | 0.832 | 6 (9.14) | 28 (90.32) | 1.000 |
| clindamycine | Yes | 20 (0.06) | 5 (4.76) |  | 64 (98.92) | 3 (9.68) |  |
| vancomycine | No | 331 (0.98) | 98 (93.33) | 0.024 | 1 (1.08) | 30 (96.77) | 0.544 |

Variables significantly associated with opium use or ICU admission were incorporated into the propensity score matching (PSM) to assess the influence of opium use on ICU admission. Categorical variables were presented as frequency (%), and chi square tests were used for the comparisons (weighted chi square tests were used for matched data). Numeric variables were reported as mean ± standard deviation (SD), and compared using the Mann-Whitney U test (weighted Mann-Whitney U test was used for matching data). P-values of less than 0.05 were considered statistically significant.

**Table S3**. Patients’ characteristics, comorbidities, treatments, and laboratory tests before and after propensity score matching [For outcome = Mortality]

| **Variable** | **Level** | **Before Propensity Score-Matched patients** | | | **After Propensity Score-Matched patients** | | |
| --- | --- | --- | --- | --- | --- | --- | --- |
|  |  | **Non-user Opium (n=337)** | **User Opium (n=105)** | **P-value** | **Non-user Opium (n=65)** | **User Opium (n=31)** | **P-value** |
| Age | ---- | 80.48 ± 5.70 | 59.59 ± 16.49 | 0 | 77.58 ± 4.29 | 74.35 ± 14.96 | 0.915 |
| Tempreture | ---- | 36.27 ± 6.06 | 37.16 ± 0.69 | 0.63 | 37.34 ± 0.91 | 37.16 ± 0.73 | 0.654 |
| BP | ---- | 128.62 ± 22.26 | 123.51 ± 21.74 | 0.024 | 126.72 ± 19.26 | 122.00 ± 27.65 | 0.333 |
| PLT | ---- | 212.09 ± 90.08 | 237.10 ± 105.67 | 0.018 | 217.86 ± 76.67 | 220.31 ± 83.88 | 0.699 |
| NUT | ---- | 67.23 ± 22.17 | 66.18 ± 25.10 | 0.801 | 65.76 ± 24.96 | 64.30 ± 30.05 | 0.782 |
| LYMP | ---- | 20.80 ± 12.64 | 19.99 ± 13.25 | 0.36 | 19.92 ± 11.91 | 18.98 ± 18.72 | 0.194 |
| CRP | ---- | 39.38 ± 48.12 | 48.29 ± 69.73 | 0.833 | 54.52 ± 59.25 | 61.09 ± 96.19 | 0.665 |
| INR | ---- | 1.20 ± 0.88 | 1.14 ± 0.24 | 0.397 | 1.11 ± 0.19 | 1.11 ± 0.12 | 0.718 |
| FBS | ---- | 151.84 ± 84.99 | 160.92 ± 104.21 | 0.579 | 165.78 ± 89.26 | 166.00 ± 107.90 | 0.856 |
| BUN | ---- | 37.90 ± 43.63 | 35.24 ± 43.20 | 0.079 | 50.11 ± 51.19 | 49.00 ± 57.02 | 0.683 |
| LDL | ---- | 77.00 ± 37.07 | 87.08 ± 76.86 | 0.881 | 81.49 ± 30.38 | 75.78 ± 33.85 | 0.458 |
| AST | ---- | 76.51 ± 288.87 | 127.01 ± 488.37 | 0.952 | 109.52 ± 419.31 | 62.15 ± 45.91 | 0.507 |
| Time_ventilation | ---- | 0.72 ± 1.82 | 1.38 ± 2.57 | 0.010 | 1.08 ± 2.00 | 1.08 ± 2.04 | 0.719 |
| Outcome_time | ---- | 12.43 ± 8.00 | 12.40 ± 9.66 | 0.446 | 14.37 ± 9.85 | 12.19 ± 6.84 | 0.571 |
| Sex | Male | 175 (0.52) | 92 (0.88) | <0.001 | 43 (71.79) | 20 (76.92) | 0.792 |
| Sex | Female | 162 (0.48) | 13 (0.12) |  | 17 (28.21) | 6 (23.08) |  |
| Smoking | Non-smoker | 311 (0.92) | 32 (0.30) | <0.001 | 38 (63.46) | 18 (69.23) | 0.632 |
| Smoking | Smoker | 26 (0.08) | 73 (0.70) |  | 22 (36.54) | 8 (30.77) |  |
| Alcohol | Non-alkoholic | 333 (0.99) | 79 (0.75) | 0.816 | 56 (92.95) | 22 (84.62) | 0.236 |
| Alcohol | Alkoholic | 4 (0.01) | 26 (0.25) |  | 4 (7.05) | 4 (15.38) |  |
| opium | Non-user | 337 (1.00) | 0 (0.00) | 0.027 | 60 (100.00) | 0 (0.00) | 0.891 |
| opium | User | 0 (0.00) | 105 (1.00) |  | 32 (53.21) | 26 (100.00) |  |
| Bad_breath | No | 154 (0.46) | 50 (0.48) | <0.001 | 28 (46.79) | 15 (57.69) | 1.000 |
| Bad_breath | Yes | 183 (0.54) | 55 (0.52) |  | 58 (96.15) | 11 (42.31) |  |
| Cancer | No | 329 (0.98) | 97 (0.92) | 0.003 | 2 (3.85) | 25 (96.15) | 0.693 |
| Cancer | Yes | 8 (0.02) | 8 (0.08) |  | 55 (92.31) | 1 (3.85) |  |
| Ghalyan_history | No | 330 (0.98) | 86 (0.82) | 0.736 | 5 (7.69) | 23 (88.46) | 1.000 |
| Ghalyan_history | Yes | 7 (0.02) | 19 (0.18) |  | 36 (59.62) | 3 (11.54) |  |
| Ventilator_need | No | 288 (0.85) | 76 (0.72) | 1.000 | 24 (40.38) | 15 (57.69) | 1.000 |
| Ventilator_need | Yes | 49 (0.15) | 29 (0.28) |  | 56 (93.59) | 11 (42.31) |  |
| Airbroncogram | No | 309 (0.92) | 98 (0.93) | 0.028 | 4 (6.41) | 24 (92.31) | 0.809 |
| Airbroncogram | Yes | 28 (0.08) | 7 (0.07) |  | 21 (34.62) | 2 (7.69) |  |
| Bilateralinvolvment | No | 110 (0.33) | 34 (0.32) | 1.000 | 39 (65.38) | 10 (38.46) | 1.000 |
| Bilateralinvolvment | Yes | 227 (0.67) | 71 (0.68) |  | 56 (93.59) | 16 (61.54) |  |
| ceftazidime | No | 302 (0.90) | 102 (0.97) | 0.024 | 4 (6.41) | 25 (96.15) | 1.000 |
| ceftazidime | Yes | 35 (0.10) | 3 (0.03) |  | 55 (92.31) | 1 (3.85) |  |
| meropenem | No | 307 (0.91) | 95 (0.90) | 1.000 | 5 (7.69) | 24 (92.31) | 1.000 |
| meropenem | Yes | 30 (0.09) | 10 (0.10) |  | 59 (98.72) | 2 (7.69) |  |
| vancomycine | No | 331 (0.98) | 98 (0.93) | 0.983 | 1 (1.28) | 26 (100.00) | 0.516 |
| vancomycine | Yes | 6 (0.02) | 7 (0.07) |  | 59 (98.72) | 0 (0.00) |  |
| linezolid | No | 329 (0.98) | 103 (0.98) | 0.983 | 1 (1.28) | 25 (96.15) | 0.270 |
| linezolid | Yes | 8 (0.02) | 2 (0.02) |  | 51 (84.62) | 1 (3.85) |  |

Variables significantly associated with opium use or mortality were incorporated into the propensity score matching (PSM) to assess the influence of opium use on mortality. Categorical variables were presented as frequency (%), and chi square tests were used for the comparisons (weighted chi square tests were used for matched data). Numeric variables were reported as mean ± standard deviation (SD), and compared using the Mann-Whitney U test (weighted Mann-Whitney U test was used for matching data). P-values of less than 0.05 were considered statistically significant.

**Table S4**. Patients’ characteristics, comorbidities, treatments, and laboratory tests before and after propensity score matching [For outcome = Need to Intubation]

| **Variable** | **Level** | **Before Propensity Score-Matched patients** | | | **After Propensity Score-Matched patients** | | |
| --- | --- | --- | --- | --- | --- | --- | --- |
|  |  | **Non-user Opium (n=337)** | **User Opium (n=105)** | **P-value** | **Non-user Opium (n=60)** | **User Opium (n=26)** | **P-value** |
| Age | ---- | 80.48 ± 5.70 | 59.59 ± 16.49 | <0.001 | 77.58 ± 4.29 | 74.35 ± 14.96 | 0.915 |
| Tempreture | ---- | 36.27 ± 6.06 | 37.16 ± 0.69 | 0.63 | 37.34 ± 0.91 | 37.16 ± 0.73 | 0.654 |
| BP | ---- | 128.62 ± 22.26 | 123.51 ± 21.74 | 0.024 | 126.72 ± 19.26 | 122.00 ± 27.65 | 0.333 |
| PLT | ---- | 212.09 ± 90.08 | 237.10 ± 105.67 | 0.018 | 217.86 ± 76.67 | 220.31 ± 83.88 | 0.699 |
| NUT | ---- | 67.23 ± 22.17 | 66.18 ± 25.10 | 0.801 | 65.76 ± 24.96 | 64.30 ± 30.05 | 0.782 |
| LYMP | ---- | 20.80 ± 12.64 | 19.99 ± 13.25 | 0.360 | 19.92 ± 11.91 | 18.98 ± 18.72 | 0.194 |
| CRP | ---- | 39.38 ± 48.12 | 48.29 ± 69.73 | 0.833 | 54.52 ± 59.25 | 61.09 ± 96.19 | 0.665 |
| INR | ---- | 1.20 ± 0.88 | 1.14 ± 0.24 | 0.397 | 1.11 ± 0.19 | 1.11 ± 0.12 | 0.718 |
| FBS | ---- | 151.84 ± 84.99 | 160.92 ± 104.21 | 0.579 | 165.78 ± 89.26 | 166.00 ± 107.90 | 0.856 |
| BUN | ---- | 37.90 ± 43.63 | 35.24 ± 43.20 | 0.079 | 50.11 ± 51.19 | 49.00 ± 57.02 | 0.683 |
| LDL | ---- | 77.00 ± 37.07 | 87.08 ± 76.86 | 0.881 | 81.49 ± 30.38 | 75.78 ± 33.85 | 0.458 |
| AST | ---- | 76.51 ± 288.87 | 127.01 ± 488.37 | 0.952 | 109.52 ± 419.31 | 62.15 ± 45.91 | 0.507 |
| Time_ventilation | ---- | 0.72 ± 1.82 | 1.38 ± 2.57 | 0.010 | 1.08 ± 2.00 | 1.08 ± 2.04 | 0.719 |
| Outcome_time | ---- | 12.43 ± 8.00 | 12.40 ± 9.66 | 0.446 | 14.37 ± 9.85 | 12.19 ± 6.84 | 0.571 |
| Sex | Male | 175 (0.52) | 92 (0.88) |  | 43 (71.79) | 20 (76.92) |  |
| Sex | Female | 162 (0.48) | 13 (0.12) |  | 17 (28.21) | 6 (23.08) |  |
| Smoking | Non-smoker | 311 (0.92) | 32 (0.30) | <0.001 | 38 (63.46) | 18 (69.23) | 0.333 |
| Smoking | Smoker | 26 (0.08) | 73 (0.70) |  | 22 (36.54) | 8 (30.77) |  |
| Alcohol | Non-alkoholic | 333 (0.99) | 79 (0.75) | <0.001 | 56 (92.95) | 22 (84.62) | 0.699 |
| Alcohol | Alkoholic | 4 (0.01) | 26 (0.25) |  | 4 (7.05) | 4 (15.38) |  |
| opium | Non-user | 337 (1.00) | 0 (0.00) | 0.816 | 60 (100.00) | 0 (0.00) | 0.782 |
| opium | User | 0 (0.00) | 105 (1.00) |  | 32 (53.21) | 26 (100.00) |  |
| Bad_breath | No | 154 (0.46) | 50 (0.48) | 0.027 | 28 (46.79) | 15 (57.69) | 0.194 |
| Bad_breath | Yes | 183 (0.54) | 55 (0.52) |  | 58 (96.15) | 11 (42.31) |  |
| Cancer | No | 329 (0.98) | 97 (0.92) | <0.001 | 2 (3.85) | 25 (96.15) | 0.665 |
| Cancer | Yes | 8 (0.02) | 8 (0.08) |  | 55 (92.31) | 1 (3.85) |  |
| Ghalyan_history | No | 330 (0.98) | 86 (0.82) | 0.003 | 5 (7.69) | 23 (88.46) | 0.718 |
| Ghalyan_history | Yes | 7 (0.02) | 19 (0.18) |  | 36 (59.62) | 3 (11.54) |  |
| Ventilator_need | No | 288 (0.85) | 76 (0.72) | 0.736 | 24 (40.38) | 15 (57.69) | 0.856 |
| Ventilator_need | Yes | 49 (0.15) | 29 (0.28) |  | 56 (93.59) | 11 (42.31) |  |
| Airbroncogram | No | 309 (0.92) | 98 (0.93) | 1.000 | 4 (6.41) | 24 (92.31) | 0.683 |
| Airbroncogram | Yes | 28 (0.08) | 7 (0.07) |  | 21 (34.62) | 2 (7.69) |  |
| Bilateralinvolvment | No | 110 (0.33) | 34 (0.32) | 0.028 | 39 (65.38) | 10 (38.46) | 0.458 |
| Bilateralinvolvment | Yes | 227 (0.67) | 71 (0.68) |  | 56 (93.59) | 16 (61.54) |  |
| ceftazidime | No | 302 (0.90) | 102 (0.97) | 1.000 | 4 (6.41) | 25 (96.15) | 0.507 |
| ceftazidime | Yes | 35 (0.10) | 3 (0.03) |  | 55 (92.31) | 1 (3.85) |  |
| meropenem | No | 307 (0.91) | 95 (0.90) | 0.024 | 5 (7.69) | 24 (92.31) | 0.719 |
| meropenem | Yes | 30 (0.09) | 10 (0.10) |  | 59 (98.72) | 2 (7.69) |  |
| vancomycine | No | 331 (0.98) | 98 (0.93) | 1.000 | 1 (1.28) | 26 (100.00) | 0.571 |
| vancomycine | Yes | 6 (0.02) | 7 (0.07) |  | 59 (98.72) | 0 (0.00) |  |
| linezolid | No | 329 (0.98) | 103 (0.98) | 0.983 | 1 (1.28) | 25 (96.15) | 0.513 |
| linezolid | Yes | 8 (0.02) | 2 (0.02) |  | 51 (84.62) | 1 (3.85) |  |

Variables significantly associated with opium use or need to intubation were incorporated into the propensity score matching (PSM) to assess the influence of opium use on need to intubation. Categorical variables were presented as frequency (%), and chi square tests were used for the comparisons (weighted chi square tests were used for matched data). Numeric variables were reported as mean ± standard deviation (SD), and compared using the Mann-Whitney U test (weighted Mann-Whitney U test was used for matching data). P-values of less than 0.05 were considered statistically significant.
